# Social capital and the COVID-19 pandemic threat: The Russian experience

**DOI:** 10.3389/fsoc.2022.957215

**Published:** 2022-12-14

**Authors:** Alexander Tatarko, Tomas Jurcik, Klaus Boehnke

**Affiliations:** ^1^National Research University Higher School of Economics, Moscow, Russia; ^2^Constructor University, Bremen, Germany

**Keywords:** social capital, social relationships, social cohesion, social trust, institutional trust, perceived coronavirus threat

## Abstract

Social capital is an important resource for the wellbeing of both the individual and society. Since the beginning of the COVID-19 pandemic, many studies have been conducted to explore the role of social capital in coping with the negative consequences of the pandemic. However, how the pandemic itself can affect the social capital of people has yet to be studied. Try to fill this gap, we aimed at testing the association between the individually perceived coronavirus threat and such indicators of social capital as general social trust, institutional trust, and the quality of various types of people's social relationships (with family, friends, colleagues, neighbors, residents of a locality, residents of a country). Data were collected in different regions of the Russian Federation for a convenience sample of 500 respondents. The study found that the individually perceived coronavirus threat was positively associated with institutional trust, but not with general social trust. Moreover, this covariation was moderated by age: an institutional trust-threat relation emerged only in older respondents with an average age of around 60, but not in younger participants. Furthermore, the study found that perceived coronavirus threat was associated with closer relationships in the family, but simultaneously with an increased distance in relations with neighbors and residents of the respondents' locality. In summary, the study indicated that “strong” ties (i.e., with family, colleagues, and friends) either remained unchanged or were intensified in the face of the pandemic threat, whereas “weak” ties (i.e., with neighbors, residents of the same locality, and fellow citizens) tended to weaken even more.

## Introduction

Government responses to the COVID-19 pandemic have involved significant restrictions in social contact as a result of externally-imposed mass quarantines and lockdowns. However, isolation has also emerged on an individual level due to fears of contracting COVID-19 (Jurcik et al., 2020; Moccia et al., 2020). The COVID-19 lockdowns have been associated with numerous financial economic stressors, physical and mental health concerns (e.g., Baker and Wilson, 2020; Jurcik et al., 2020; Pandey et al., 2020; Joffe, 2021). It has also altered the relationships with people as they become increasingly physically, and sometimes socially, distant from each other. The current study examines the experience of living through the coronavirus pandemic as it relates to the psychosocial phenomena that form the basis of social capital at the macrolevel (i.e., social and institutional trust) as well as the microlevel (e.g., relationships with others—relatives, colleagues, neighbors). We view the perceived COVID-19 threat as a psychological indicator of the impact of the pandemic and resulting lockdowns. The perceived threat implies that the individual makes a subjective assessment of the phenomenon and the perceived likelihood that an event will occur and will have specific consequences (Agrawal, 2018; Wirtz and Rohrbeck, 2018). An understanding of how the experience of living with the threat of viral infection can be associated with the components of social capital is crucial, first, to assess the expected consequences of the current pandemic and, second, to predict the consequences of future pandemics.

### Social capital

The concept of social capital is frequently discussed in the social and economic sciences (sociology, social psychology, political science, and economics) and can be defined in a variety of ways. Social capital comprises not only a cognitive component (i.e., norms of reciprocity and trust) but also a relational component (i.e., social relationships and networks). In almost all definitions and studies, social capital involves trust and social ties or social relationships (Bourdieu, 1986; Coleman, 1988; Putnam, 2000; Lin, 2001). Putnam (2000, p. 19) defines social capital as “connections among individuals–social networks and the norms of reciprocity and trustworthiness that arise from them.” He argues that trust is an essential component of social capital because it modifies cooperation. This study also considers social capital with regard to trust (social and institutional) and social relationships (e.g., with relatives, friends, colleagues).

Theoretically, social capital should be considered as a micro concept whereas social cohesion, being a broader concept than social capital, is a more appropriate concept for macro analysis (Klein, 2013). Therefore, data at the individual level should be used only to analyze the relationship between the indicators of social capital and other phenomena. However, the results obtained also allow us to make inferences about what would happen to social cohesion, since the concepts of social capital and social cohesion are closely intertwined.

The well-known concept of social cohesion (Dragolov et al., 2016; Delhey et al., 2018) includes in its structure three components evaluated at the macro-level of society: *Connectedness* (identification, institutional trust, perception of fairness), *social relations* (social networks, trust in people, acceptance of diversity), and *focus on the common good* (solidarity and responsibility for others, respect for social rules, and civil participation). In our study, we measure the following indicators of social capital at the individual level: institutional trust, social trust, and perceived dynamics of social relations. Thus, the indicators of social capital that we measured are associated with indicators of social cohesion, allowing us to make some generalizations.

### Social capital and health

Most studies in this field examine the relationship between social capital and economic progress (Helliwell and Putnam, 1995; Knack and Keefer, 1997; Fukuyama, 2002). However, numerous studies strongly indicate that social capital is positively associated with human health (Kawachi et al., 1997, 1999; Macinko and Starfield, 2001). Social capital is linked to health through several different causal pathways, for example through a rapid circulation of health information, healthy norms, access to material resources, lower crime rates, and emotional support in networks (Rönnerstrand, 2013).

### Social capital and COVID-19

Social capital is a resource that can help prevent the spread of COVID-19. Based on US data, it was found that individuals reduced their mobility earlier and to a higher degree in counties with high levels of social capital than in counties with low levels of social capital (Borgonovi and Andrieu, 2020). Thus, in counties with high social capital, people more effectively shared information about the perceived danger of the virus, trusted this information and reduced their mobility, thereby preventing infections.

According to empirical research conducted in various European countries (in independent analyses for Austria, Germany, Italy, the Netherlands, Sweden, Switzerland and the UK) between March and May 2020, higher social capital accounted for a 12–32% reduction in the incidence of COVID-19. Moreover, in Italy, areas with higher social capital exhibited a lower mortality from COVID-19 (Bartscher et al., 2020). This can primarily be attributed to the fact that high social capital increases social responsibility: people maintain social distancing, observe lockdowns, wear protective equipment, and follow the recommendations of the government, public health officials, and physicians. Relatedly, people in such areas may generally be more supportive of others (e.g., checking in on ill neighbors).

Of course, whether some of these interventions, including mass quarantines, are necessarily in the best interest of the public is hotly contended by laypeople and scientists alike (Bavli et al., 2020; Jurcik et al., 2020; Reiss and Bhakdi, 2020). The lockdowns and mass vaccination campaigns themselves were controversial and have been associated with various negative outcomes (e.g., Joffe, 2021), and thus there is reason for people to be discerning with respect to trusting the advice of government authorities. Lockdowns that were organized in many countries of the world after the outbreak of the pandemic restrained its spread. On the other hand, public health interventions also had numerous negative consequences for the economy and also on the unwanted physical and mental health impacts on the population at large (Bavli et al., 2020). For instance, some people may have developed symptoms of depression and anxiety from the social isolation, while others may have delayed needed medical care for chronic illnesses such as cancer or cardiovascular diseases due to the fear of contracting the virus (e.g., Bavli et al., 2020; Jurcik et al., 2020). In this regard, the pandemic situation may negatively affect various components of social capital. For example, focusing on others as potential sources of viral transmission and the ensuing and enforced social distancing may lead to the weakening of affective bonds within a community.

In other words, social capital can be used effectively for the public good and even misused by authorities. Thus, studying the impact of the pandemic threat on social capital may provide us with insights into how and which types of individual and public health interventions are accepted by the community, which interventions may increase social capital and which types may erode it.

### Aims and research questions of the present study

The purpose of our study was to understand how the perceived threat of the coronavirus can be associated with various aspects of social capital at the individual level.

Accordingly, we can formulate two main research questions.

RQ1. How (positively or negatively) is the perceived threat of the coronavirus related to (general and institutional) trust?
RQ2. How is the perceived threat of the coronavirus related to various types of social relationships (with relatives, neighbors, colleagues, etc.)? Are people beginning to distance from each other or not?

### Impact of the COVID-19 pandemic on social capital

The available body of survey evidence demonstrates that national disasters, including epidemics adversely influence social capital (Albrecht, 2017). Concerns have already been expressed that the COVID-19 pandemic can have negative consequences for social capital (Pitas and Ehmer, 2020). Measures such as isolation and social distancing taken to contain the virus can contribute to the destruction of social capital. The daily interaction with different people that takes place in everyday life at work, school and in public places was stopped or minimized during the pandemic. Past pandemics of a similar nature have had negative implications for social capital. Having studied the effects of the pandemics from the Spanish flu of 1918 to COVID-19, some authors have argued that the Spanish Flu pandemic had negative consequences on social trust (Aassve et al., 2020). Moreover, a low level of social trust was inherited by the descendants, which only exacerbated and slowed economic development for many decades (Aassve et al., 2020). The decline in trust was the result of the measures taken to combat the pandemic: social isolation, closure of public places, a ban on mass meetings, and a request by the authorities to avoid interpersonal contacts. Similar restrictive measures were taken during the COVID-19 pandemic, so we can generally expect a negative effect of the pandemic on people's trust.

In response to the COVID-19 pandemic, rumors have circulated regarding the alleged man-made nature of COVID-19 (Shukhratovna et al., 2021). These theories about how the pandemic emerged contributed to the growth of xenophobia and fears of a digital dictatorship, which took the form of protests on social networks against applications of monitoring the population's compliance with social isolation (Shukhratovna et al., 2021). These “conspiracy theories” may not always be unfounded; for instance, the Chinese government reportedly had used their COVID tracking app to disperse potential protesters in early 2022 who had aspects of their bank deposits frozen (Jung, 2022). Ultimately these beliefs and actions can also have an adverse effect on institutional and social trust and they can contribute to feelings of alienation as well. In other words, the more the COVID-19 pandemic is perceived as dangerous and threatening, and the more severe and extensive the lockdowns, the less social capital we would expect there to be. However, even more complex is the relationship between the perceived coronavirus threat and the dimensions of social capital at the individual level: different types of trust, such as general trust and institutional trust, as well as attitudes concerning specific social contacts. One large international study indicated that confinement during the pandemic triggered reductions in social activity with neighbors, friends, and family, which in turn was associated with reduced life satisfaction (Ammar et al., 2020). Thus, we expect that a perceived coronavirus threat and the associated restriction of social contacts may have negative consequences for social capital. In particular, social ties between people and institutions will not be maintained and will become weaker, as a result of the above-mentioned fears, people's trust may decrease. Based on this reasoning, we can formulate our first hypothesis:

*H1:* The perceived threat of coronavirus will be negatively associated with social capital (social and institutional trust, social relationships with others).

On the other hand, it is important to note that the COVID-19 pandemic is different from pandemics in the past: we now have advanced communication and information technologies available at our fingertips. Staying at home no longer means near complete isolation. We can work, study, even see family and friends as well as our physicians and therapists online, which became commonplace during the pandemic (Jurcik et al., 2020).

Nevertheless, does this alternative digital form of communication negate or buffer the threat to social capital? Scientists argue that today there are many ambiguities regarding the use of digital technology in new realities, as people may be uncertain about how to use them appropriately and effectively. Moreover, digital communication is not an equivalent substitute for personal interaction (Claridge, 2020; Pitas and Ehmer, 2020). However, it is reasonable to expect that the use of Information and Communication Technology (ICT) can mitigate the decline in social capital. Analogously, there is evidence that long-distance psychotherapies can be as effective as therapies that are delivered face-to-face (Carlbring et al., 2018), even though digitally guided expert treatments may not be the preferred modality by the public (Renn et al., 2019). Such findings beg the question as to whether being able to communicate *via* social media, email, text, and online platforms such as Zoom or Skype, can afford a level of social capital that can be as effective as communicating in person, at least for some people.

Moreover, there is the evidence that disasters can, perhaps paradoxically, strengthen social capital (Dussaillant and Guzman, 2015). Dussaillant and Guzman (2015) found that in some cases trust increased after an earthquake and tsunami; disasters influence people's attitudes, behavior and social norms, and thus provide an opportunity to strengthen their social ties. Boehnke et al. (1989) found that higher fear of nuclear war among West German adolescents covaried positively with self-reported wellbeing. Similarly, the pandemic has led to new opportunities for social connections and collaboration, where some people may make an extra effort to connect with colleagues on projects online, even across multiple continents (see Jurcik et al., 2020). Indeed, despite some of the negative effects on socialization during the mass quarantines there was a significant increase in social contacts through digital technology according to one large scale international study (Ammar et al., 2020). The authors suggested wide scale interventions would promote social inclusion through technology. Thus, the question about how the pandemic affects social capital remains open and the present study contributes to empirically addressing this issue.

Accordingly, an alternative hypothesis can also be proposed that posits that the perception of the coronavirus threat does not reduce social capital but even increases it, because ICT opens up new opportunities for people to contact and interact with each other. In addition, in lockdown conditions, contacts with significant others, for example, with some family members and close friends, can become even more intense and frequent.

*H2:* The perceived threat of the coronavirus will be positively associated with social relationships with others.

Consequently, we are faced with contradicting suppositions regarding the relationship between the perceived threat of the coronavirus and social capital. This study aims to resolve this contradiction. Moreover, previous studies examining the relationship between the COVID-19 pandemic and social capital have been focused on how social capital helps combat the spread of the pandemic. This study examines the opposite side of the issue—namely, how the pandemic might influence social capital. Additionally, while previous studies have examined the pandemic in connection with social capital at the macrolevel, the current study emphasizes social capital at the individual level.

## Methods

### Procedure

The empirical study was conducted at the height of the COVID-19 pandemic, in May 2020, when the lockdown (first officially introduced in Russia on March 25, 2020) was extended by the Russian government until early June 2020 and the restrictions on movement had not yet been lifted. The study was conducted online through a paid online survey service called “Anketolog” (https://anketolog.ru).

### Participants

Five hundred participants took part in the study in exchange for compensation (about 6 USD per respondent). The sample included 32.8% men and 67.2% women. The characteristics of the respondents' age are as follows: *M*age = 38.5, *SD*age = 10.66, Min_age_ = 18, Max_age_ = 70. The full age distribution of respondents is shown in the histogram in Appendix. Most of the respondents (72%) reported having a higher education, 5.4% had secondary education, and 19% had secondary special education (vocational schools, colleges). As for material status, 7% of respondents live on their income without experiencing material difficulties; 45.2% said that their income is quite enough for them; 32.6% said that it was difficult for them to live on their income; 14% reported financial difficulties, i.e., that it was very difficult for them to live on their income; 1.2% of respondents found it difficult to assess their material status. Twenty-four percent of respondents resided in Moscow and the Moscow region, and the remaining 76% respondents resided in other regions of the Russian Federation.

### Materials

All measures were administered in Russian. The questionnaire contained the translated measures shaped by back-translation and cognitive interviews with the think-aloud technique (Willis, 2004).

### Perceived coronavirus threat

We used the Perceived Coronavirus Threat Questionnaire (PCTQ) (Conway et al., 2020). The questionnaire contains 6 items, such as “Thinking about the coronavirus (COVID-19) makes me feel threatened,” “I am worried that I or people I love will get sick from the coronavirus (COVID-19).” We used the following responses on a 5-point Likert scale: (1) completely disagree; (2) disagree; (3) not sure/neutral; (4) somewhat agree; (5) completely agree. The Cronbach alpha of the Russian version of the questionnaire was 0.87.

### Social trust

We assessed social trust using 4 statements. Three of them are taken from the World Values Survey questionnaire: “Most people can be trusted,” “I trust my neighbors,” “I trust people of other nationalities” (Inglehart et al., 2014). The forth was developed by the authors (“I trust my colleagues at work”). The 5-point Likert scale had the following response options: (1) completely disagree; (2) disagree; (3) not sure/neutral; (4) somewhat agree; (5) completely agree. The Cronbach's alpha for this scale was 0.83.

### Institutional trust

We assessed institutional trust using four statements, developed by the authors: “I trust the federal authorities,” “I trust the regional authorities,” “I trust the authorities of the city/district in which I live,” “I trust the mass media.” We used the following responses on a 5-point scale: (1) completely disagree; (2) disagree; (3) not sure/neutral; (4) somewhat agree; (5) completely agree. Cronbach alpha was 0.91.

### Social relationships

We evaluated the respondents' social relationships with various groups of individuals: family members, friends, colleagues, neighbors, residents of the same locality (city, town, village) and Russian population (as a whole). Respondents were asked: “How did the COVID-19 situation affect your relationship with…?” The sentence was completed with the list of representatives of the above-mentioned social categories from family members to residents of the same state. Respondents were offered a 5-point Likert-type scale, to evaluate whether there was a distancing in the relationship or a greater closeness: (1) Has certainly contributed to distancing, (2) probably contributed to distancing, (3) the relationship has not changed, (4) likely contributed to the greater closeness, (5) definitely contributed to greater closeness. The six targets of social relationships were treated separately in the subsequent analyses.

### Control variables

We used five additional control variables. Three were demographics: education, age and gender. The variable “education” included 11 levels in accordance with the increase in the degree of education. These stages corresponded to the official Russian classification of education stages from 1 - Basic secondary education to 11 - Academic degree stage II – PhD. The variable “age” was continuous. Respondents had to indicate their age, measured by the number of full years. The variable “gender” was categorical and coded as follows: 1 – male; 2 – female.

Given the topic of the current research, we also asked participants to document their personal experiences with COVID-19. Firstly, we asked the respondents: “Have you ever had a coronavirus infection?” (Response options: 1 = yes, 0 = no). We further asked the respondents whether people they knew had experienced the infection: “Do you have any friends or relatives who have been or are currently suffering from a coronavirus infection?” (Response options: 1 = yes, 0 = no).

### Data processing

For data processing, we first constructed an intercorrelation matrix (Spearman coefficient) and calculated the descriptive statistics. To assess the relationship between perceived coronavirus threat, trust, and social relationships, we used linear regression analysis controlling for socio-demographic characteristics, as well the respondents' own experiences with the coronavirus. Linear regression analysis was performed in the SPSS program, reporting standardized regression coefficients. Additionally, we performed a moderation analysis using PROCESS (Hayes, 2013) in SPSS to determine whether there are interactions with age for some of the relations we identified. Age was used a moderator given that older age groups are at a greater mortality risk (Mishra et al., 2020).

## Results

Table 1 presents descriptive statistics and a correlation matrix. To comment briefly on the resulting correlations, it should be noted that the perceived COVID-19 threat is positively associated with institutional trust, closer relationships with family members, and more distant relationships with neighbors and other local residents. Social trust is positively associated with institutional trust as well as closer relationships with neighbors and other local residents. In addition to being positively associated with the perceived threat of the coronavirus and social trust, institutional trust is also positively associated with closer relationships with family members, friends, and colleagues. Furthermore, all types of social relations were found to be more or less related to each other.

**Table 1:** Descriptive statistics and correlations between variables.

**Variable**	**M**	**SD**	**1**	**2**	**3**	**4**	**5**	**6**	**7**	**8**	**9**
1. Virus threat	3.26	0.98	1	0.00	0.17***	0.13**	−0.03	−0.08	−0.12**	−0.14**	−0.06
2. Social trust	2.90	0.81		1	0.41***	0.05	0.079	0.08	0.14**	0.12**	0.11*
3. Institutional trust	2.24	0.94			1	0.13**	0.15***	0.10*	0.02	0.07	0.06
4. Family	3.27	0.91				1	0.27***	0.09*	0.14**	0.16***	0.14***
5. Friends	2.78	0.79					1	0.50***	0.51***	0.48***	0.37***
6. Colleagues	2.74	0.74						1	0.47***	0.38***	0.31***
7. Neighbors	2.81	0.62							1	0.58***	0.40***
8. Residents of the town/village	2.70	0.72								1	0.71***
9. Russian population (as a whole)	2.70	0.78									1

*p < 0.05; **p < 0.01; ***p < 0.001.

Table 2 presents the results of a multiple regression analysis of the correlation between the perceived threat of the coronavirus and social and institutional trust with controlled sociodemographic variables. In addition, there were two other important control variables that might affect the components of social capital: the presence/absence of COVID-19 patients among the acquaintances and relatives of the respondents and whether or not the respondents themselves had contracted the virus. Standardized β coefficients are presented in the following tables.

**Table 2:** Relation among perceived coronavirus threat, social and institutional trust (simple linear regression with control of demographic variables, *N* = 500).

**Predictor and controls**	**Social trust (Model 1)**				**Institutional trust (Model 2)**			
	**β**	**t**	**SE**	**95% CI**	**β**	**t**	**SE**	**95% CI**
Virus threat (predictor)	0.01	0.03	0.04	−0.06–0.08	0.17***	3.72	0.04	0.08–0.25
Education	0.00	0.04	0.06	−0.11–0.12	−0.02	−0.49	0.07	−0.17–0.10
Low material status	−0.11*	−2.32	0.04	−0.18 to −0.01	−0.17***	−3.71	0.05	−0.28 to −0.09
Age	0.23***	5.09	0.00	0.01–0.02	0.10*	2.03	0.00	0.00–0.02
Sex	−0.04	−0.88	0.08	−0.22–0.08	0.07	1.59	0.09	−0.03–0.33
Sick personally	−0.03	−0.63	0.16	−0.42–0.22	−0.10*	−2.25	0.19	−0.79 to −0.05
Friends/relatives sick	0.09	1.89	0.08	−0.01–0.31	0.11*	2.48	0.09	0.04–0.41

Model 1: F = 5.18, df = 5, *p* < 0.00; R^2^ = 0.07; Effect sizes (Cohen's f^2^) is 0.08.

Model 2: F = 5.63, df = 5, *p* < 0.001; R^2^ = 0.08; Effect sizes (Cohen's f^2^) is 0.09.

*p < 0.05; **p < 0.01; ***p < 0.001.

The perceived threat of the coronavirus was not associated with social trust but was positively associated with institutional trust: the greater the perceived threat of the coronavirus in the eyes of the respondents, the greater their reported trust in the various levels of basic governmental institutions and the media. Among the reference variables, both types of trust are negatively associated with low material status and positively associated with the age of the respondents. With regard to institutional trust, there was also significance in whether or not respondents had acquaintances or relatives with COVID-19 (positive correlations) and whether respondents themselves had reported having been infected (negative relation).

Similarly, Table 3 presents models for relations (degree of closeness or distance since the beginning of the pandemic) with family members and friends.

**Table 3:** Relation between perceived coronavirus threat and perceived closeness with family and friends (simple linear regression with control of demographic variables, *N* = 500).

**Predictor and controls**	**Family (Model 3)**				**Friends (Model 4)**			
	**β**	**t**	**SE**	**95% CI**	**β**	**t**	**SE**	**95% CI**
Virus threat (predictor)	0.13**	2.73	0.03	0.03–0.20	−0.02	−0.48	0.04	−0.09–0.06
Education	0.06	1.40	0.07	−0.04–0.23	−0.06	−1.20	0.06	−0.19–0.05
Low material status	−0.12*	−2.53	0.05	−0.22 to −0.03	−0.10*	−2.21	0.04	−0.18 to −0.01
Age	0.01	0.22	0.01	−0.00–0.01	0.08	1.69	0.01	−0.01–0.013
Sex	0.02	0.61	0.09	−0.12–0.24	0.01	0.27	0.08	−0.14–0.18
Sick personally	−0.04	−0.91	0.19	−0.54–0.19	−0.05	−1.17	0.16	−0.51–0.13
Friends/relatives sick	−0.06	−1.46	0.09	−0.33–0.05	0.08	1.64	0.08	−0.03–0.29

Model 3: F = 2.62, df = 5, *p* = 0.002; R^2^ = 0.05; Effect sizes (Cohen's f^2^) is 0.05.

Model 4: F = 1.59, df = 5, *p* = 0.14; R^2^ = 0.02 Effect sizes (Cohen's) is 0.02.

*p < 0.05; **p < 0.01; ***p < 0.001.

The perceived threat of the coronavirus was unrelated to the perceived closeness with friends but was positively related to the perception of closer relationships with family members. Among the reference variables, only a negative correlation between low material status and the perception of closer relationships with family and friends was found.

Table 4 reveals that there is no relation between the perceived COVID-19 threat and the perceived closeness in relationships with colleagues, but there is a negative relation with the perception of relationships with neighbors.

**Table 4:** Relation between perceived coronavirus threat and perceived closeness with colleagues and neighbors (simple linear regression with control of demographic variables, *N* = 500).

**Predictor and controls**	**Colleagues (Model 5)**				**Neighbors (Model 6)**			
	**β**	**t**	**SE**	**95% CI**	**β**	**t**	**SE**	**95% CI**
Virus threat (predictor)	−0.06	−1.36	0.04	−0.12–0.02	−0.12**	−2.64	0.03	−0.13 to −0.02
Education	−0.04	−0.82	0.05	−0.16–0.07	−0.06	−1.38	0.05	−0.16–0.03
Low material status	−0.06	−1.31	0.04	−0.13–0.03	−0.07	−1.60	0.03	−0.12–0.01
Age	0.05	1.02	0.01	−0.01–0.01	0.11*	2.38	0.01	0.01–0.02
Sex	0.02	0.39	0.08	−0.12–0.18	−0.01	−0.10	0.06	−0.13–0.12
Sick personally	−0.02	−0.48	0.16	−0.38–0.23	−0.10*	−2.21	0.13	−0.53 to −0.03
Friends/relatives sick	0.005	0.10	0.08	−0.14–0.16	0.08	1.69	0.06	−0.02–0.23

Model 5: F = 0.73, df = 5, *p* = 0.64; R^2^ = 0.01; Effect sizes (Cohen's f^2^) is 0.01.

Model 6: F = 3.34, df = 5, *p* = 0.002; R^2^ = 0.05; Effect sizes (Cohen's f^2^) is 0.05.

*p < 0.05; **p < 0.01; ***p < 0.001.

Additionally, in Model 6 (neighbors), statistically significant relations with the dependent variable have two reference variables: the age of respondents was positively associated with the perception of closeness with neighbors, and having ever personally contracted the disease was negatively associated with this dependent variable.

Table 5 indicates that the perceived threat of the coronavirus was in no way associated with the perception of relationship closeness with the population of the country at large (model 8).

**Table 5:** Relation between perceived coronavirus threat and perceived closeness with the participants' town/village and Russian population (as a whole) (simple linear regression with control of demographic variables, *N* = 500).

**Predictor and controls**	**Participants' town/village (Model 7)**				**Russian population (as a whole) (Model 8)**			
	**β**	**t**	**SE**	**95% CI**	**β**	**t**	**SE**	**95%F CI**
Virus threat (predictor)	−0.13**	−2.82	0.03	−0.16 to −0.03	−0.04	−0.93	0.04	−0.10–0.04
Education	−0.07	−1.65	0.06	−0.20–0.02	−0.04	−0.80	0.06	−0.17–0.07
Low material status	−0.11*	−2.40	0.04	−0.17 to −0.02	−0.12*	−2.50	0.04	−0.19 to −0.02
Age	0.03	0.57	0.01	−0.01–0.01	0.01	0.15	0.03	−0.01–0.01
Sex	−0.02	−0.38	0.07	−0.17–0.11	−0.05	−0.99	0.08	−0.23–0.08
Sick personally	−0.03	−0.59	0.15	−0.38–0.20	−0.01	−0.31	0.16	−0.37–0.27
Friends/relatives sick	0.08	1.61	0.07	−0.03–0.26	0.01	0.28	0.08	−0.13–0.18

Model 7: F = 3.09, df = 5, *p* = 0.006; R^2^ = 0.04; Effect sizes (Cohen's f^2^) is 0.04.

Model 8: F = 0.82, df = 5, *p* = 0.52; R^2^ = 0.01; Effect sizes (Cohen's f^2^) is 0.01.

*p < 0.05; **p < 0.01; ***p < 0.001.

However, we obtained a negative correlation between the perceived threat of the coronavirus and the perception of closeness in relationships with other residents of the locality in which the respondents live. In other words, the higher the threat of the coronavirus, the more the respondents report being alienated from other local residents. Moreover, among the control variables, only low material status was negatively associated with the participants' town/village and Russian population (as a whole). This indicates that the higher the material status of the respondents, the stronger the feeling of closeness with residents of the same locality as well as the country as a whole.

Given that age is a risk factor for coronavirus infection and also proved to be associated with the dependent variable in several cases (Models 1, 2, and 6), we evaluated the moderating role of age with regard to the relationship between the threat of the coronavirus and social capital indicators. A moderating effect was only discovered in relation to one case: the association between institutional trust and coronavirus threat (model 2). The moderating effect had the following characteristics: effect = 0.11, *p* < 0.05; 95 CI = 0.02 to 0.20; *F* (3, 495) = 7.42, *p* < 0.001.

**Figure 1:**
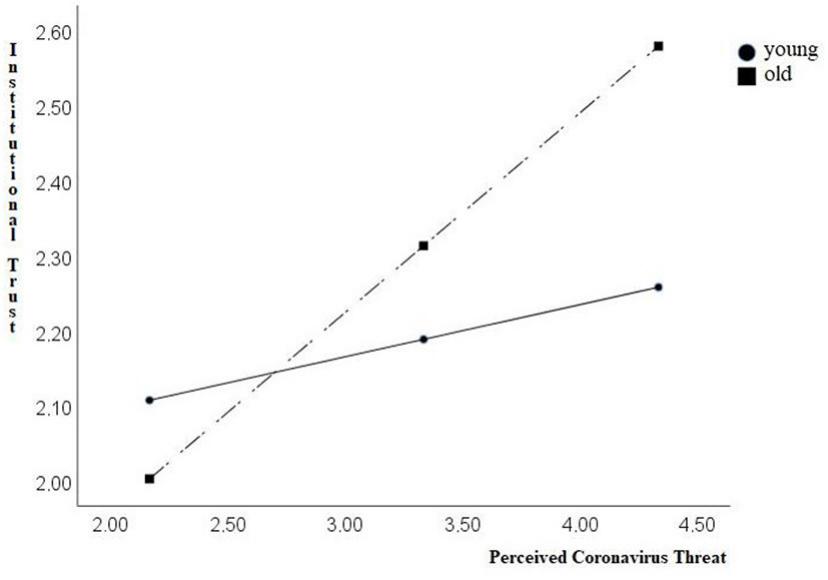
Graphical depiction of the interaction effect between perceived coronavirus threat (X) and age for institutional trust (Y) (*N* = 500).

Figure 1 provides a visual representation of this interaction effect.

Thus, institutional trust is low when the threat is low, regardless of age. When the threat is high, the level of institutional trust among younger respondents remains practically unchanged, while the slope was steeper among older respondents. We thus analyzed conditional effects of the focal predictor at values of the moderator. We found that for the group of young people (16th percentile, 29 years old), the effect of perceived coronavirus threat on institutional trust failed to reach significance (Effect = 0.07, ns; SE = 0.06; *t* = 1.92; 95 CI: = −0.04 to 0.19). In contrast, for the group of older participants (84th percentile, 50 years old) the effect of perceived coronavirus threat on institutional trust was significant (Effect = 0.28, *p* < 0.001; SE = 0.06; *t* = 4.31; 95 CI: = 0.15 to 0.40).

## Discussion

In this study, we examined the effect of the perceived coronavirus threat on various aspects of social capital at the individual level. We suggested two competing hypotheses, given some of the mixed anecdotal and empirical evidence. The first was that the perceived threat of coronavirus would be negatively associated with social capital (social and institutional trust, social relationships with others). The second, was that the perceived threat of the coronavirus would be positively associated with social relationships with others.

However, as with most research studies, reality proved more complex: negative relations between COVID-19 threat and social capital were found for relationships with neighbors and local residents. In contrast, positive relations were obtained between the perceived coronavirus threat and institutional trust and relationships with relatives. However, there were no associations with other indicators, including social trust, relationships with friends, colleagues, or closeness with fellow citizens.

Social capital is strengthened/accumulated in certain spheres from which people can receive support in the face of the viral threat: family and the state. That is, participants with higher levels of perceived threat reported greater closeness with family (which might also be a consequence of lockdowns and constant cohabitation) and a higher level of loyalty to the state (through institutional trust). However, greater institutional trust was only observed in the group most vulnerable to COVID-19—namely, respondents around 60 years of age.

With regard to social ties, we see a general process of disintegration. Respondents endorsed a greater closeness within the family but simultaneously more distancing from members of other social categories (neighbors, residents of the same locality), while relationships with colleagues and friends, as with fellow citizens, reportedly remained unchanged. However, the social category of “fellow citizens” (i.e., the Russian population at large) may be too abstract, and, perhaps, respondents are simply unable to assess their own relationships with members of this category. Overall, we see that so-called “strong” ties (with family, friends, and colleagues) or relationships with those with whom the respondents are in close contact remain unchanged or became stronger. Meanwhile, “weak” ties (with neighbors or residents of the same locality) or ties with those with whom the respondents may on average have less contact with have become reportedly even weaker. From our point of view, all these effects are precisely a reaction to threat and isolation. Family ties become stronger because people worry about their next of kin and contact them more often in isolation. As for weak ties, people begin to contact them less due to the COVID-19 threat and isolation, so these ties become weaker.

If we consider the results of our study from the broader macro perspective of social cohesion (Dragolov et al., 2016, 2018), it is likely that the impact of the pandemic on social cohesion will be uneven. If trust is not particularly affected by the pandemic, then certain aspects of social relations may suffer and people may move away from each other.

Findings that clarify the link between fear of COVID-19 and social capital are extremely important as numerous studies conducted since the onset of the COVID-19 pandemic have indicated that social capital itself is an important resource for overcoming the disease (Bian et al., 2020; Barrios et al., 2021; Makridis and Wu, 2021). Notably, the findings also demonstrated that those who reported having been infected with the virus also endorsed less institutional trust. The reason for this finding is unclear, but for most people (especially those without underlying health conditions) a course of COVID-19 does not lead to severe complications, which may contrast with some of the messages from public health authorities and the media; these have often focused on statistical models with overly negative population outcomes, or on salient outlying cases, generating considerable controversy in the public and scientific circles alike (see Reiss and Bhakdi, 2020). This begs the question as to whether such messaging may sometimes be counter-productive. More research needs to be done on public health campaigns and perceptions of the virus in those that have been infected compared to those who have not.

## Strengths and limitations

This is the first empirical study that attempts to consider the effects of the perceived coronavirus threat on social capital. We analyzed the relationship between the perceived coronavirus threat and social capital at two levels. First, at the individual level, the psychological phenomena that form social capital at the macro-level (institutional and social trust) were considered. Second, we considered the respondent's subjective assessment of changes in relationships with others (relatives, colleagues, neighbors, etc.), which constitutes social capital at the individual- or at the micro-level.

However, the peculiarities of the relationship between the perceived threat of the coronavirus and social capital may depend on the prevailing situation in the country and, primarily, on the effectiveness of the state's efforts in overcoming the pandemic. For example, if these efforts are ineffective, the substantial threat posed by the pandemic may adversely affect institutional trust. Therefore, to further appreciate the role of contextual elements, this study could be conducted in other countries and cultural settings. Replication studies will facilitate an understanding of the universality of the relationship between integration and disintegration processes in various societies amidst the pandemic.

At a basic diagnostic level, when we asked respondents whether they or their acquaintances had experienced a coronavirus infection or not, we did not require that the disease or its absence be necessarily documented with a positive or negative test result, respectively. Therefore, we can assume that the sample may include a certain number of people who answered these questions in the affirmative, based on their impressions, which may be incorrect (i.e., false positives) given symptom overlap with other viral infections such as the flu (see Kaye et al., 2020). Alternatively, some respondents may have answered these questions in the negative, the disease could have still progressed in an asymptomatic or mild form (i.e., false negative). Indeed, asymptomatic cases may be fairly common according to an epidemiological study (Kim et al., 2020). What complicates this diagnostic picture further is that the medical tests themselves (e.g., PCR tests) have limitations in sensitivity and specificity (Jarrom et al., 2022).

Additionally, our study does not have a longitudinal design; although we asked about perceived changes in retrospect, respondents' impressions may be susceptible to various recall biases and cognitive heuristics. Accordingly, we cannot assume causality or even the direction of causal relationships. In some cases, the reverse logic of explaining the connection may also be plausible. For example, greater perceived coronavirus threat may be a consequence of the respondents' inherently high institutional trust, but the reverse may also be true: health anxious individuals may look for answers in authoritative-sounding sources of reassurance and guidance. Bidirectional or looping effects are also possible: those who trust institutions may trust the official state reports about the coronavirus danger and experience a higher perceived threat, which in turn may make them more dependent on and trusting of the government authorities to solve the pandemic. Similarly, those who reported having been infected with the virus also reported less institutional trust, but it is unclear which variable caused the other, or if a third variable was involved in affecting this relation.

Another limitation of our study was that the effect sizes in only two regression models out of eight is close to the average. The effect sizes in our models are mostly low. Nevertheless, the effect of the coronavirus threat on social capital exists and should not be underestimated.

Finally, the data collected for this study arises from a convenience sample. Therefore, it may help us gain insights into the considered phenomena, but we are not able to generalize this data to the Russian population as a whole.

## Conclusion

The vast majority of available research on social capital in the context of COVID-19 shows that social capital is a good resource for mitigating the rise in morbidity and preventing the spread of infection (see the research review by Wu, 2021). However, researchers overlook the fact that the pandemic itself can be associated with psychosocial phenomena underlying social capital (i.e., various types of trust and social relationships). This study intended to fill this particular gap.

The results of the study demonstrate that greater perceived coronavirus threat was linked to higher institutional trust in older participants. This pattern can be interpreted as the activation of psychological defense mechanisms. This effect was not observed among young people, for whom the infection is less dangerous (Bonanad et al., 2020). In contrast, the perceived threat of the coronavirus was not related to social trust. As for social ties, our study indicated that “strong” ties (with family, colleagues, and friends) either remained unchanged or were intensified in the face of the epidemiological threat. “Weak ties” (with neighbors, residents of the same locality, and fellow citizens) have tended to weaken even more. Accordingly, the possible effects of the pandemic on social capital are ambiguous and may impact various parameters of social capital in differential ways. We observed social disintegration combined with a growth in paternalism and increased ties with the immediate social environment. Therefore, some might conclude that the social cohesion of Russian society has suffered somewhat as a result of the pandemic. Overall, our findings suggest more of a negative effect on social cohesion than a neutral one, even if not all ties were adversely affected. In addition to replication studies, future research needs to examine the relations between public health and media messaging, numerous pandemic related health indicators in society (other than COVID outcomes per se), and institutional trust.

## Data availability statement

The original contributions presented in the study are publicly available. This data can be found here: AT. (2021). Social capital and COVID [Data set]. Zenodo. http://doi.org/10.5281/zenodo.4814352.

## Ethics statement

All procedures performed in the studies involving human participants were in accordance with the ethical standards of the Commission Ethical Assessment of Empirical Research Projects at HSE Department of Psychology and with the 1964 Helsinki Declaration and its later amendments or comparable ethical standards.

## Author contributions

AT designed the study, collected, and processed the data. AT, TJ, and KB were involved in the process of writing the paper. All authors contributed to the article and approved the submitted version.

## Figures and Tables

**Figure A1:**
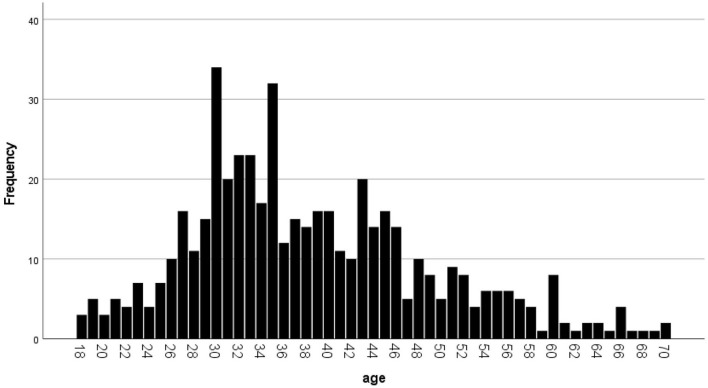
Age distribution of the sample (*N* = 500).
